# Deletion of 82–85 N-Terminal Residues in SARS-CoV-2 Nsp1 Restricts Virus Replication

**DOI:** 10.3390/v16050689

**Published:** 2024-04-26

**Authors:** Gianni Gori Savellini, Gabriele Anichini, Fabrizio Manetti, Claudia Immacolata Trivisani, Maria Grazia Cusi

**Affiliations:** 1 Department of Medical Biotechnologies, University of Siena, 53100 Siena, Italy; gabriele.anichini2@unisi.it; 2 Department of Biotechnology, Chemistry and Pharmacy, University of Siena, 53100 Siena, Italyclaudia.trivisani2@unisi.it (C.I.T.)

**Keywords:** Nsp1, attenuated viruses, transcription and translation, host shutdown, leader sequence, SARS-CoV-2

## Abstract

Non-structural protein 1 (Nsp1) represents one of the most crucial SARS-CoV-2 virulence factors by inhibiting the translation of host mRNAs and promoting their degradation. We selected naturally occurring virus lineages with specific Nsp1 deletions located at both the N- and C-terminus of the protein. Our data provide new insights into how Nsp1 coordinates these functions on host and viral mRNA recognition. Residues 82–85 in the N-terminal part of Nsp1 likely play a role in docking the 40S mRNA entry channel, preserving the inhibition of host gene expression without affecting cellular mRNA decay. Furthermore, this domain prevents viral mRNAs containing the 5′-leader sequence to escape translational repression. These findings support the presence of distinct domains within the Nsp1 protein that differentially modulate mRNA recognition, translation and turnover. These insights have implications for the development of drugs targeting viral proteins and provides new evidences of how specific mutations in SARS-CoV-2 Nsp1 could attenuate the virus.

## 1. Introduction

The CoronaVIrus Disease-19 (COVID-19) pandemic, caused by Severe Acute Respiratory Syndrome CoronaVirus 2 SARS-CoV-2, has resulted in over 774 million confirmed infections and 7 million deaths [1]. Understanding the molecular mechanisms of viral virulence factors and virus–host interactions is critical for the development of preventive and therapeutic measures against COVID-19 disease. Viruses commonly employ host translation shutoff to evade innate immune responses, primarily mediated by beta Interferon (IFN-β) [2,3,4,5]. Furthermore, several strategies are pursued by viruses in order to inhibit host protein synthesis, targeting various stages of the translation process such as mRNA transcription, processing, nuclear export of newly formed mRNAs, mRNA degradation, and blocking key translation and elongation factors [2,3,4,5,6,7,8,9,10,11,12]. Coronaviruses (CoVs), including SARS-CoV-2, induce this host shutoff through multiple viral proteins [13,14,15,16]. SARS-CoV-2 virion is composed of the positive-sense RNA (vRNA) genome and several structural proteins (N, M, E, and Spike) [17,18,19,20,21]. The vRNA genome also encodes sixteen non-structural proteins that form the replication machinery and seven accessory proteins [17,18,19,20,21]. Although the exact functions of SARS-CoV-2 accessory proteins are not fully demonstrated, previous studies suggest that these proteins are not essential for viral replication but they can modulate virus pathogenesis interacting with host pathways, especially those involved in innate immunity. Indeed, SARS-CoV-2 is also known to encode several viral proteins that antagonize interferons (IFNs) and interferon-stimulated genes (ISGs), and hence promote infection and early virus replication [6,22,23,24,25,26,27,28,29,30,31,32]. Coronaviruses generate a set of sub-genomic RNAs (sgRNAs) through a discontinuous transcription mechanism, likely influenced by negative-sense RNA and a conserved 5′-leader sequence of about 70 nucleotides [33,34,35,36]. These sgRNAs encode structural, non-structural, and accessory viral proteins. When the translation of cellular mRNAs is impaired, the viral mRNAs become prioritized, enhancing viral protein synthesis, assembly, and release [37,38,39]. Notably, SARS-CoV-2 Nsp1 has been extensively studied in this context. It directly binds to the 40S ribosomal subunit, obstructing the mRNA entry channel, thus reducing cellular protein synthesis [39]. In our study, we analyzed the genetic fitness of SARS-CoV-2 strains having naturally occurring deletions within the Nsp1 protein. We assessed the impact of these Nsp1 deletion mutants on both host cellular gene and viral mRNAs translation. Specifically, we identified deletions at the N-terminus (aa: 82–85 and 82–86) and the C-terminus (aa: 141–143) of Nsp1 in human samples. Our findings highlight a conserved domain in the N-terminal region (a.a. 82–85) of Nsp1 that primarily impedes cellular mRNA translation rather than influencing mRNA decay. Indeed, the Δ82–85 and Δ82–86 Nsp1 variants showed reduced degrading activity towards GFP expression driven by the human beta-globin promoter at the transcriptional level (mRNA). Moreover, the Δ82–85/Δ82–86 Nsp1 variant displayed decreased efficiency in recognizing viral mRNAs, making these transcripts susceptible to translational inhibition rather than decay. Therefore, this mechanism could potentially reduce SARS-CoV-2 virulence by limiting viral gene expression. Indeed, the specific activities of Δ82–85 and Δ82–86 Nsp1 mutants were further evident as they showed a marked reduction in virus fitness and replication, in both interferon-deficient (Vero E6) and interferon-competent (Calu-3) cells. The specific Nsp1 domain identified in this study opens the way to drug development, targeting the Nsp1 virulence factor as a potential therapeutic approach for infections.

## 2. Materials and Methods

### 2.1. Cell Cultures and Virus Isolates

Vero E6 (ATCC CRL-1586), human embryonic kidney HEK-293T (ATCC CRL-3216), and Calu-3 (ATCC HTB-55) cells were maintained in Dulbecco’s modified Eagle’s medium (DMEM) (Euroclone, Milan, Italy). This medium was supplemented with 100 U/mL penicillin/streptomycin and 10% heat-inactivated fetal bovine serum (FCS) (Euroclone) and incubated at 37 °C with 5% CO_2_. Selected clinical specimens of Omicron and BQ1.1 lineages underwent Whole Genome Sequencing using the COVIDSeq test (Illumina, Milan, Italy). The sequenced genomes have been deposited in GenBank under the accession numbers: MT531537, ON974845, OR166017, OR166015, OR160428, and OR161044. In accordance with the Declaration of Helsinki principles (approved by the Ethics Committee under BIOBANK-MIU-2010, with Amendment No. 1 dated 17 February 2020), and after obtaining written, signed, and dated informed consent from participants at the Virology Laboratory of Santa Maria alle Scotte Hospital in Siena (Italy), the identified SARS-CoV-2 lineages were isolated using Vero E6 cells. These isolates were subsequently stored at −80 °C in individual aliquots and viral titers were determined using the Median Tissue Culture Infectious Dose (TCID50/mL) assay.

### 2.2. Plasmids

Viral genomic RNA was extracted using the QIAamp Viral RNA Kit (Qiagen, Milan, Italy). HA-tagged full-length Nsp1 gene fragments were synthesized via Reverse-Transcription Polymerase Chain Reaction (RT-PCR) using the SuperScript™ III One-Step RT-PCR System with Platinum™ Taq High-Fidelity DNA Polymerase (Life Technologies, Milan, Italy). Subsequently, these fragments were cloned into the EcoR1-XhoI restriction sites of the pCAGGS-MCS plasmid following standard procedures. All constructed plasmids underwent sequence verification through Sanger sequencing. The constructs containing the human β-globin 5′-UTR, or the SARS-CoV-2 5′-UTR and the Leader fused with green fluorescent protein, were generously provided by Prof. Michal Schwartz from the Weizmann Institute of Science, Rehovot, Israel.

### 2.3. Kinetics of Viral Replication

Monolayers of Vero E6 and Calu-3 cells were seeded at densities of 1 × 10^5^ and 2 × 10^5^ cells per well, respectively, in 24-well plates using complete culture medium. The following day, the cultures were infected with a multiplicity of infection (MOI) of 0.01 of the selected SARS-CoV-2 lineages and incubated at 37 °C for 1 h. After the incubation, the inoculum was carefully removed and the cells were extensively washed. Subsequently, growth medium containing 2% FBS was added to the wells. The cytopathic effect (CPE) was assessed under a light microscope three days post-infection. Supernatants from the infected cultures were harvested at 24, 48, and 72 h (h) post-infection (p.i.) and stored at −80 °C for further analyses. Viral release from the infected cultures was quantified using a virus microtitration assay on Vero E6 cells plated in 96-well plates. Viral titers were calculated using the Reed–Muench formula and expressed as TCID_50_/_mL_.

### 2.4. Assessment of IFN-β Expression

Medium from Calu-3 cells, infected as previously described, was collected at 48, 72 h post-infection (p.i.). IFN-β quantification was assessed by VeriKine-HS Human IFN Beta TCM ELISA Kit (PBL assay science, Piscataway, NJ, USA), following manufacturer’s instructions. The results are presented as mean fold values relative to the mock-infected sample ± standard deviation from at least three independent experiments.

### 2.5. Western Blotting

HEK-293T cells co-transfected with HBB-, 5′-Ld-SL1- or 5′-UTR-GFP, and Nsp1 constructs were harvested, and cell pellets were lysed using RIPA buffer supplemented with an anti-protease cocktail from Roche, Milan, Italy. Total protein content in the lysates was quantified using the BCA assay from Pierce (Milan, Italy). A total of 25 µg of proteins from each sample was prepared in Laemmli sample buffer, denatured by boiling for 5 min, and then separated by SDS-PAGE. The resolved proteins were transferred onto nitrocellulose membranes (Santa Cruz Biotechnology, Heidelberg, Germany). After blocking with 5% non-fat dry milk, membranes were probed with primary antibodies: anti-GFP (Life Technologies, Milan, Italy), anti-HA tag (Merk-Millipore), or anti-GAPDH (Life Technologies) as a loading control. Following three washes with PBS-T (phosphate-buffered saline containing 0.05% Tween-20), the membranes were incubated with an HRP-conjugated anti-mouse IgG secondary antibody (Merck-Millipore) at room temperature for 1 h. The immunoreactive bands were visualized by using the TMB-Blotting 1-Step Solution (Pierce).

### 2.6. Reverse-Transcription Quantitative PCR (RT-qPCR)

HEK-293T cells (1 × 10^5^/well) were plated in 24-well plates and, after O/N incubation, cell monolayers were transfected with 200 ng of either HBB-, 5′-Ld-SL1-, or 5′-UTR-GFP expressing plasmids in combination with 500 ng of empty vector, Wuhan-1, or mutant Nsp1 plasmids. Cells were collected at 48 h post-transfection and total RNA was isolated using the RNAeasy Plus mini kit (Qiagen, Milan, Italy). TaqPath 1-Step Multiplex Master Mix (Thermo Fisher Scientific, Milan, Italy) was used for RT-qPCR reactions in a QuantStudio 5 Real-Time PCR System (Thermo Fisher Scientific). TaqMan^®^ Assays (Thermo Fisher Scientific) for GFP and actin (ACT) were used for specific transcripts detection. Each sample was run in duplicate and the cycle threshold (Ct) values of each gene were normalized against the endogenous ACT gene and compared with the negative, empty vector transfected, control. The results were represented as mean fold relative increments from at least three independent experiments (2^−ΔΔCt^ algorithm) ± standard deviations (SDs). Where indicated, cells were treated with 100 μg/mL of cycloheximide (CHX) (Merck-Millipore, Milan, Italy), 5 μg/mL of actinomycin D (ActD) (Life Technologies, Milan, Italy) at 48 h post-transfection or left untreated and collected at the starting point (T0), 2 h and 4 h post-treatment. Samples were processed for RT-qPCR as described above or lysed in RIPA buffer for subsequent immunoblotting.

### 2.7. Cycloheximide Chase Analysis and NSs Protein Stability

Vero E6 cells were seeded in 24-well plates and infected as previously described. Forty-eight hours post-infection, cells were collected for starting protein expression quantification (T0), while the remaining samples were treated with 100 μg/mL of cycloheximide (CHX). Samples were then collected 2 h and 4 h later for time course quantification. The amount of Nsp1 and GAPDH loading control proteins was quantified by Western blotting and densitometric analysis using the JmageJ 1.53t software. Relative Nsp1 intensities were normalized with respect to the corresponding GAPDH signal value. Fold changes of each sample were calculated relative to the corresponding T0 mock-treated sample.

### 2.8. Computational Details

The protein structure of the Nsp1 N-terminal domain was taken from the Protein Data Bank using the accession code 7K7P. The Δ82–85 protein structure was built with the Residue and Loop mutation tool available in Maestro using the Nsp1 N-terminal domain (7K7P) as a template. The 5′-Ld-SL1 mRNA belonging to the viral 5′-UTR was extracted from the PDB entry 2GDT. All the structures were prepared using the Protein Preparation Wizard, which added missing side chains and hydrogens, assigned bond orders, and minimized hydrogen atoms. Docking was conducted using the HDOCK server. Visual analyses were performed using PyMol Molecular Graphics System (version 2.5.0).

### 2.9. Statistics

Mean differences were statistically analyzed by using one-way ANOVA with Dunnett’s multiple comparison test in GraphPad Prism 6 (GraphPad Software, San Diego, CA, USA), in order to compare prevalence rates among different study groups. Statistical significance was set at *p* < 0.05.

## 3. Results

### 3.1. SARS-CoV-2 Strains Bearing Nsp1 N-Terminal Deletions Are Attenuated in Replication

**Figure 1:**
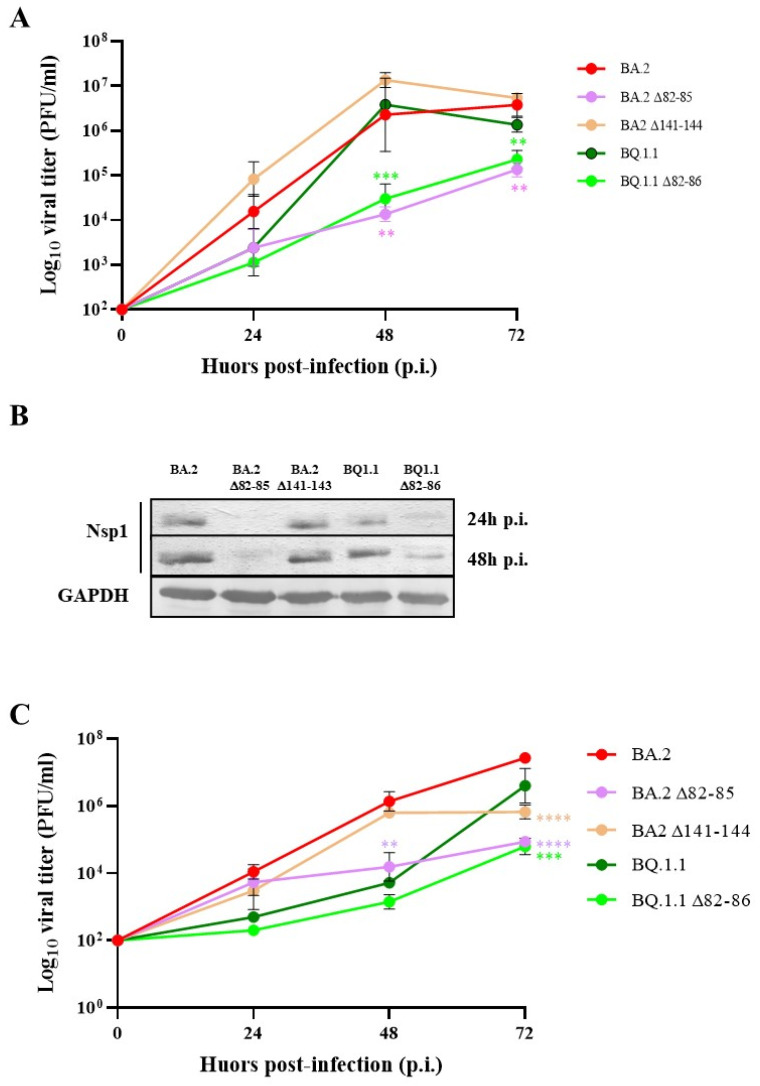
Kinetic growth of SARS-CoV-2 lineages bearing Nsp1 partial deletion. (**A**) Vero E6 cells were infected with Omicron BA.2 and the relative ∆82–85 and ∆141–143 lineages, or with the BQ.1.1 and the ∆82–86 variants at a multiplicity of infection (MOI) of 0.01. Cell culture supernatants were collected at 24 h, 48 h, and 72 h p.i. and viable progeny virus content was assessed by a microtitration assay. The results are presented as Log_10_ of the mean viral titer expressed as plaque forming units (PFUs) ± standard deviations (SDs) from at least three separate experiments. (**B**) The expression of Nsp1 was determined by Western blotting on 50 µg of whole cell lysates (WCLs) of Vero E6 cells infected with the indicated virus lineages and collected at 24 h and 48 h p.i. The loading control is represented by the immuno-detection of Glyceraldehyde 3-phosphate dehydrogenase (GAPDH) protein. (**C**) The kinetic growth of the previously described SARS-CoV-2 lineages was also investigated in type I interferon-competent Calu-3 cells. Cell culture supernatants were collected at the indicated time points and assessed for virus replication by microtitration assay. The results are plotted as Log_10_ of the mean PFU ± standard deviations (SDs) from at least three separate experiments. Significance is reported as ** *p* < 0.001, *** *p* < 0.0005, and **** *p* < 0.0001 with respect to the parental strains. On the contrary, the deletion of the 141–143 domain located at the C-terminus of BA.2 Nsp1 did not affect virus replication in Vero E6 cells, leading to virus titers similar to the parental (BA.2) strain (*p* > 0.05) (Figure 1A). Meanwhile, cells infected with original SARS-CoV-2 lineages (BA.2 and BQ1.1) and with the Δ141–143 virus variant showed similar levels of Nsp1 protein over time. Indeed, at 24 h and 48 h p.i., Nsp1 was substantially expressed by these virus lineages, while a different behavior was observed in CoV2-Δ82–85- and CoV2-Δ82–86-deleted viruses, which showed a low or undetectable production of Nsp1, indicating a possible role of this non-structural protein in reducing virus progeny release (Figure 1B). Moreover, replication kinetics of selected virus lineages were investigated in Calu-3 cells, which represent a well-defined pulmonary cell system to investigate SARS-CoV-2 fitness in a human, IFN-β-competent environment. The results are comparable to those observed in Vero E6 cells, confirming that the 82–85 domain is critical for viral fitness (Figure 1C). At late times of infections, CoV2-Δ141–143 also showed a reduced growth in Calu-3 cells with respect to BA.2 (*p* < 0.0001), albeit at a lesser extent when compared to CoV2-Δ82–85 (*p* = 0.001) and CoV2-Δ82–86 (*p* = 0.0009) (Figure 1C). Additionally, it seems that the replication kinetics of these virus strains remain unaffected in IFN-β-competent cells, suggesting that the reduced virus fitness in Calu-3 cells was not a consequence of the IFN-β susceptibility antiviral activity.

To evaluate the biological functions of Nsp1 deletion on virus fitness and innate immune response to the infection, we selected SARS-CoV-2 naturally occurring virus strains bearing 82–85, 82–86, and 141–143 amino acid deletion within the Nsp1. To this aim, we assessed replication kinetics of selected SARS-CoV-2 strains in Vero E6 cells which are type I IFN-deficient and represent the gold standard for SARS-CoV-2 growth. Infections were performed at an MOI of 0.01 and supernatants were collected for virus titration by the standard microtitration method 24, 48, and 72 h p.i. All investigated virus strains did not show significant difference in progeny virus titer at 24 h p.i. (*p* > 0.05). However, replication kinetics showed a replicative disadvantage and an evident reduction in virus progeny release compared to the relative original strains (BA.2 and BQ1.1, respectively) for viruses carrying the Nsp1 82–85 or 82–86 deletion at both 48 h (*p* = 0.0027 and *p* = 0.0001) and 72 h p.i. (*p* = 0.003 and *p* = 0.001) (Figure 1A).

### 3.2. INF-β Production by Nsp1 Deletion Strains According to Different Growth Kinetics

**Figure 2:**
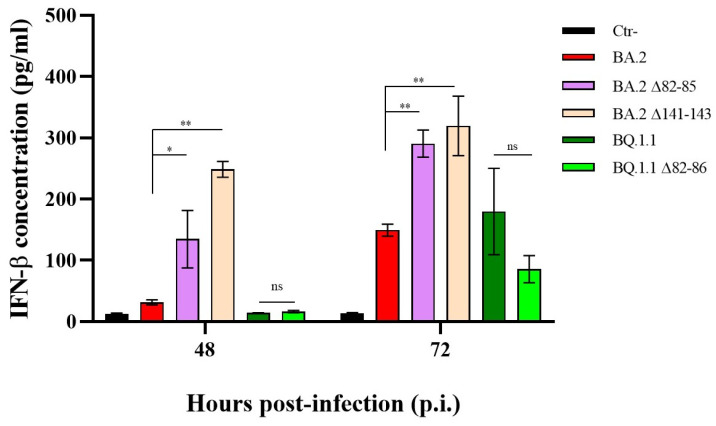
Induction of IFN-β release by partially deleted Nsp1 SARS-CoV-2 lineages. Secreted IFN-β was assessed in Calu-3 cells infected (MOI = 0.01) with BA.2 and the relative ∆82–85 and ∆141–143 lineages, or with the BQ.1.1 and the ∆82–86 variants by enzyme-linked immunoassay (ELISA) at 48 h and 72 h p.i.. Negative control (Ctr-) was represented by uninfected Calu-3 cells. Quantitative evaluation, based on relative standard curves, was performed and the results are reported as mean concentration (pg/mL) ± standard deviations (SDs) from at least three independent experiments (*n* ≥ 3). Significance is reported as ns, not significant, * *p* < 0.05 and ** *p* < 0.001 with respect to the mock-infected control (Ctr-).

To explore whether the distinct replication kinetics observed in the 82–85 and 82–86 Nsp1-deleted strains might translate to varied innate antiviral responses, we evaluated their capacity to stimulate IFN-β production in the supernatants of infected Calu-3 cells. At 48 and 72 h p.i., both the BA.2 Δ82–85- and Δ141–143-deleted variants demonstrated the ability to induce IFN-β production compared to the mock-infected control (*p* < 0.0001). Notably, the Δ141–143 variant induced higher levels of IFN-β compared to the other virus variants (Figure 2).

Conversely, the BQ.1.1 strain, along with its Δ82–86 variant, did not trigger a significant secretion of IFN-β expression at 48 h p.i (*p* > 0.05) (Figure 2). This observation was likely attributed to their limited growth in Calu-3 cells at the specified time point, as shown in Figure 1C. However, at 72 h p.i., although no significant difference was recorded between the two strains (*p* > 0.05), only the parental BQ.1.1 lineage significantly stimulated the cytokine release compared to the mock-infected control (*p* = 0.0048). These results highlight that the altered replication rates of the deleted strains do not depend on the presence of IFN-β but, mostly, reside in other strain-specific factors.

### 3.3. The N-Terminal Region of Nsp1 Emerges as Crucial for Its Interaction with the Host Cellular Transcription/Translation Machinery

**Figure 3:**
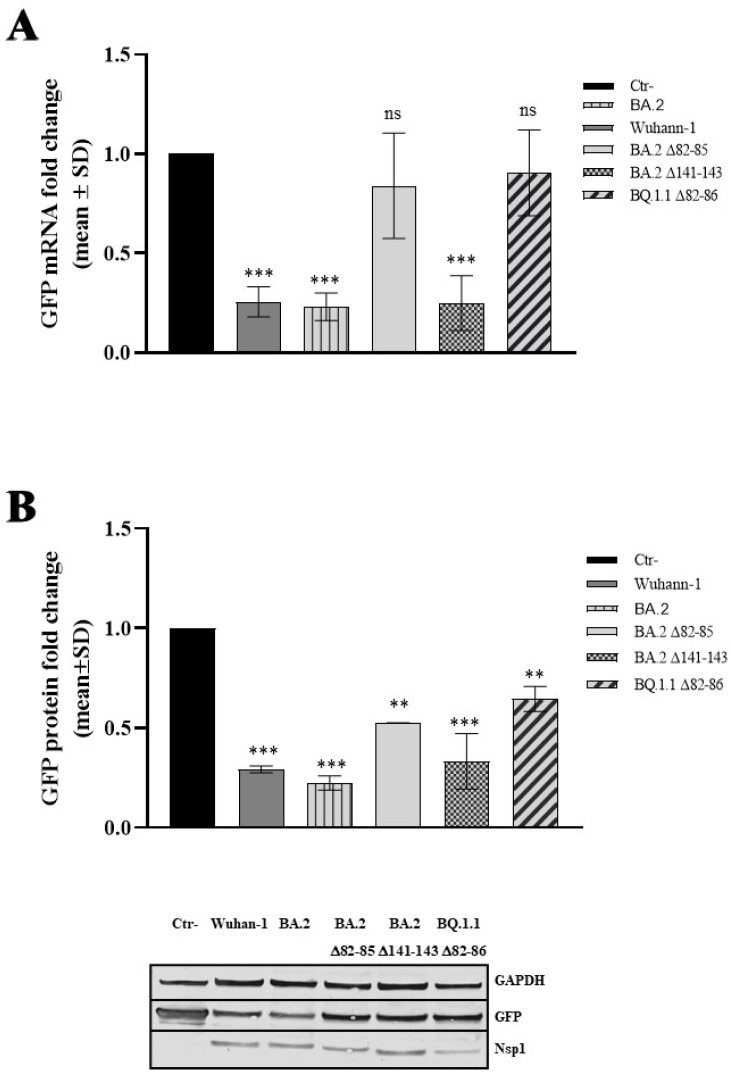
Evaluation of host cell shutoff in Nsp1 variants expressing cells. The impact of Nsp1-deleted mutants on the human beta-globin (HBB) promoter-mediated Green Fluorescent Protein (GFP) was assessed in HEK-293T cells. (**A**) The transcriptional control of GFP was examined by using total RNA purified from HBB-GFP transfected samples, either in combination with an empty plasmid (Ctr-) or Nsp1-expressing plasmids. Cells were collected at 48 h post-transfection and the specific GFP mRNA content was measured by quantitative reverse-transcription polymerase chain reaction (RT-qPCR). GAPDH gene expression served for relative quantification based on the 2^−ΔΔCt^ method. At least three independent experiments (*n* ≥ 3) were conducted, and the representative data are presented as mean values ± standard deviations. (**B**) The modulation of GFP expression in Nsp1 variants expressing HEK-293T cells was also evaluated as protein levels through Western blotting. Equal amounts of total cell lysates were resolved by SDS-PAGE, and specific antibodies were used for GFP, GAPDH, and Nsp1 proteins, with a representative image provided in the lower panel. Densitometric analysis was conducted using ImageJ software. Graph values are presented as the mean fold change in GFP band intensity ± standard deviations (SDs). Significance is reported as ‘ns’, not significant, ** *p* < 0.001 and *** *p* < 0.0005 between each sample and the control (Ctr-).

We next investigated whether the altered virus fitness was a consequence of Nsp1-modified activity. Therefore, the naturally occurring viral protein variants were cloned, and the activity of the viral proteins towards cellular and viral mRNA translation and stability was investigated. It is worth mentioning that the Nsp1 protein shares the S_135_R mutation among the Omicron sublineages with respect to the ancestral Wuhan-1; thus, only BA.2 Nsp1 was included in subsequent experiments as a control. To this purpose, HEK-293T cells, known as the gold standard for investigating human and viral protein activity through transient overexpression, were selected. HEK-293T cells were transfected to transiently express the Wuhan-1, the BA.2 or the deleted (Δ82–85; Δ82–86; and Δ141–143) Nsp1 plasmid in combination with a cellular promoter-driven (human beta globin; HBB) Green Fluorescent Protein (GFP)-expressing plasmid. GFP-specific mRNA content was estimated by quantitative RT-qPCR. As shown in Figure 3A, the RT-qPCR assay evidenced that, among the SARS-CoV-2 Nsp1 variants tested, the Wuhan-1 and BA.2 SARS-CoV-2 Nsp1 markedly suppressed HBB-mediated GFP expression at the transcriptional level, leading to a ~4-fold decrease in specific mRNA (*p* = 0.0003) compared to the basal, empty plasmid transfected sample (Ctr-).

Similarly, the Δ141–143 viral protein mutant promoted a robust HBB-driven mRNA decay, with a mean fold reduction in GFP mRNA of 3.20 ± 0.45 (*p* = 0.0007), (Figure 3A). A different behavior was instead observed in the Δ82–85 and Δ82–86 Nsp1 mutants, which displayed no degrading activity towards cellular mRNAs (*p* > 0.05) (Figure 3A). Among the Nsp1 protein mutants tested, both the Δ82–85 and Δ82–86 showed a significantly reduced degrading activity on cellular mRNAs compared to the Wuhan-1 variant, with a mean reduction in protein activity of 4.52 ± 1.43 (*p* = 0.012) and 2.65 ± 0.59 (*p* = 0.037), respectively (Figure S1A). Alteration in HBB-mediated GFP expression was also demonstrated at the protein level on lysates of cells transfected with HBB-GFP and Nsp1 variants. The GFP protein content was estimated by immunoblotting. As reported in Figure 3B, all protein variants significantly reduced reporter protein translation, although the Δ82–85 and Δ82–86 mutants had minor effects compared to the Wuhan-1 Nsp1 (Figure S1B). These results highlight the functional significance of 82–85 residues in modulating host cellular processes; moreover, they indicate that the valine residue at the 86 position (V_86_) is not a determinant for Nsp1 protein function. These insights contribute to our understanding of how SARS-CoV-2 causes cell damage and may impact on the development of therapeutic strategies targeting viral proteins.

### 3.4. The 82–85 Nsp1 N-Terminal Domain Is Involved in Viral mRNA Expression

**Figure 4:**
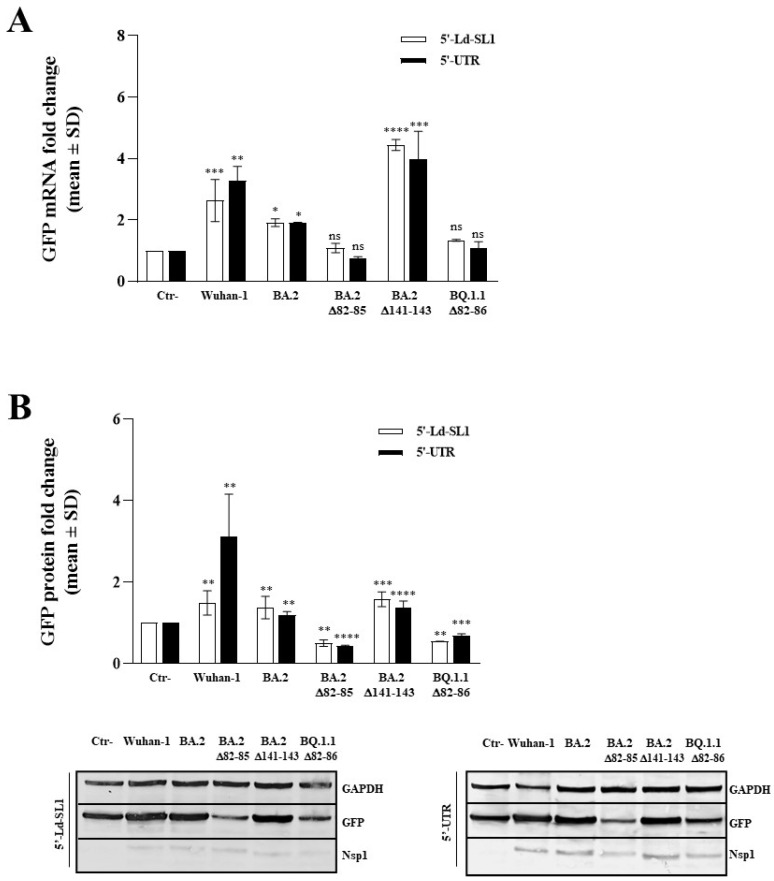
Evaluation of Nsp1 variants’ modulation of viral genes expression. The impact of Nsp1-deleted mutants on viral 5′-Ld-SL1- or 5′-UTR-mediated GFP expression was evaluated in HEK-293T cells. (**A**) Transcriptional control of GFP was examined in 5′-Ld-SL1- or 5′-UTR-GFP-transfected samples, either in combination with an empty plasmid (Ctr-) or Nsp1-expressing plasmids. Cells were collected at 48 h, and total RNA was purified. GFP and GAPDH mRNAs were quantified by RT-qPCR using the 2^−ΔΔCt^ analysis. Data were presented as mean values ± standard deviations (SD) from different experiments. (**B**) GFP protein content was assessed in Nsp1 variants expressing HEK-293T cells by Western blotting. Equal amounts of total cell lysates were resolved by SDS-PAGE, and specific antibodies were used to probe for GFP, GAPDH, and Nsp1 proteins. A representative image is provided in the lower panel. Densitometric analysis was performed using ImageJ software. Graph values are presented as the mean fold change in GFP band intensity ± standard deviations (SDs). Significance is reported as ‘ns’, not significant, * *p* < 0.05, ** *p* < 0.001, *** *p* < 0.0005, and **** *p* < 0.0001 between each sample and the control (Ctr-).

The activity of the original and deleted versions of the Nsp1 protein towards viral mRNA transcription, translation, and stability was investigated in HEK-293T cells transfected with plasmids encoding either the Wuhan-1 and BA.2 Nsp1 or the deleted versions (Δ82–85; Δ82–86, and Δ141–143) of Nsp1, along with the viral 5′-leader stem loop 1 (5′-Ld-SL1) or the full-length 5′-UTR-driven Green Fluorescent Protein (GFP). The GFP-specific mRNA content was estimated using quantitative RT-qPCR. Among the Nsp1 proteins, Wuhan-1 and BA.2 Nsp1 were capable of recognizing and mediating the expression of the reporter gene downstream of either the viral 5′-Ld-SL1 or the 5′-UTR sequences, without any evident significant difference among them (Figure S2A). An increase in GFP mRNA under the 5′-Ld-SL1 or 5′-UTR sequence was observed both in Wuhan-1 (*p* = 0.0004) and BA.2 (*p* = 0.0002), respectively (Figure 4A and Figure S2A).

Among the other investigated SARS-CoV-2 mutants, the Δ141–143 variant exhibited behavior similar to that of the Wuhan-1 Nsp1, inducing GFP mRNA accumulation using both 5′-Ld-SL1 and 5′-UTR sequences. On the contrary, the Δ82–85 and Δ82–86 Nsp1 proteins did not have any effect (*p* > 0.05) on GFP mRNA synthesis or stability (Figure 4A and Figure S2A). However, the immunoblotting performed on transfected HEK-293T cell lysates revealed an evident reduction (*p* < 0.0001) in GFP protein translation when the Δ82–85 and Δ82–86 Nsp1 proteins were expressed (Figure 4B and Figure S2B). Conversely, neither the Δ141–143 nor the Wuhan-1 Nsp1 displayed any impairment in GFP protein translation (Figure 4B and Figure S2B).

### 3.5. The 82–85 Nsp1 Domain Is Required for Cellular and Viral mRNAs Stability Control

**Figure 5:**
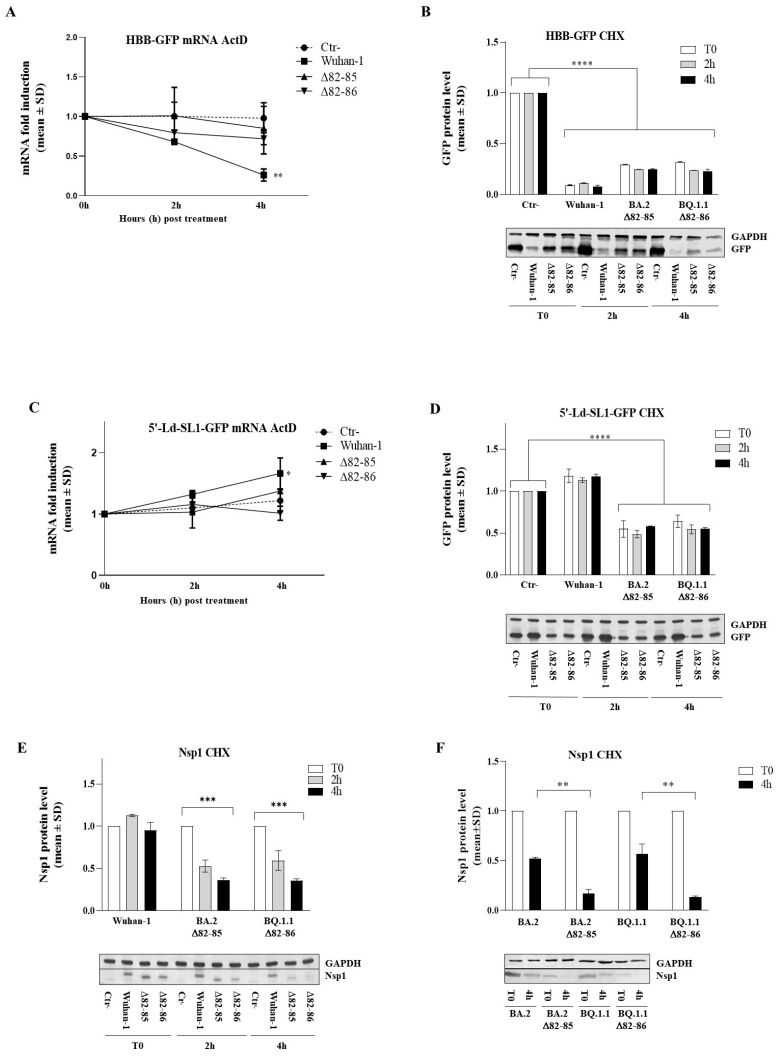
Nsp1 variants’ activity towards cellular and viral mRNAs. (**A**) The influence of both original and deleted Nsp1versions on cellular mRNA decay was investigated in HEK-293T cells. A time-course experiment was conducted using actinomycin D (ActD), a transcriptional inhibitor. Cells were collected at indicated time points, and after total RNA isolation, GFP mRNA was quantified by RT-qPCR using the 2^−ΔΔCt^ analysis. (**B**) The impact of different Nsp1 variants on HBB-mediated GFP protein expression was assessed in HEK-293T cells, either mock-treated or treated with cycloheximide (CHX). Equal amounts of total cell lysates from samples collected at indicated time points after CHX treatment were resolved by SDS-PAGE and specific antibodies were used to probe for GFP and GAPDH. (**C**) GFP mRNA levels were quantified by RT-qPCR in HEK-293T cells expressing Nsp1 variants along with either 5′-Ld-SL1- or 5′-UTR-GFP and treated with ActD for specified periods. (**D**) HEK-293T cells expressing the GFP protein downstream of either the 5′-Ld-SL1- or 5′-UTR-GFP promoters were co-transfected in order to express different Nsp1 variants. At 48 h post-transfection, cells were mock- or CHX-treated and collected at indicated times. Equal amounts of total cell lysates were resolved by SDS-PAGE and GFP or GAPDH proteins were probed by Western blotting procedure. The protein stability of Nsp1 variants was determined by Western blotting on total cell lysates of transfected HEK-293T cells (**E**) or Vero E6 cells infected with indicated virus strains (**F**) and subjected to a time-chase with CHX. Densitometric analysis of Western blotting images was performed using ImageJ software. Graph bars represent mean values ± standard deviations (SD) from different experiments. Significance is reported as * *p* < 0.05, ** *p* < 0.001, *** *p* < 0.0005, and **** *p* < 0.0001.

The results presented above highlighted the deleted Nsp1 variants’ ability to abrogate both cellular and viral protein translation without having any degrading activity towards mRNA. Based on that, we further investigated the activity of these SARS-CoV-2 Nsp1 variants in HEK-293T cells, including only the Wuhan-1 control, as BA.2 Nsp1 behaved similarly. To better understand whether the modulation of GFP protein levels previously observed in Nsp1-expressing cells resulted from altered viral protein-degrading activity of specific mRNAs, we treated transfected cells with actinomycin D (ActD), a well-known transcription inhibitor that is frequently used for mRNA stability analysis. To validate that the observed decrease in GFP protein levels in Nsp1 with HBB-GFP-expressing cells was not attributable to enhanced mRNA decay, we assessed GFP mRNA turnover following treatment with ActD. The impact of Wuhan-1 Nsp1 on GFP mRNA stability became notably pronounced over time, especially 4 h post ActD treatment (fold decrease of 0.26 ± 0.07; *p* = 0.0004). Thus, the observed drop in reporter protein content could, at least in part, be a consequence of diminished specific mRNA intracellular content (Figure 3B). Conversely, both the Δ82–85 and Δ82–86 mutants exhibited only a minimal, non-significant effect on GFP mRNA decay, as for the empty vector control (Ctr-) (*p* > 0.05) (Figure 5A). This suggests that mechanisms other than mRNA degradation occurred to inhibit protein translation.

Furthermore, the diminished GFP protein levels observed were not due to any alterations in protein stability or degradation. This conclusion was supported by the cycloheximide (CHX) chase assay to measure steady-state protein stability. CHX inhibits the elongation step in eukaryotic protein translation, thereby preventing protein synthesis upon administration. Semiquantitative immunoblotting showed that no significant decline in GFP protein levels over time (*p* > 0.05) occurred when both the Wuhan-1 and deleted Nsp1 were expressed along the reporter gene in CHX-treated HEK-293T transfected cells (Figure 5B). Intriguingly, all the aforementioned Nsp1 variants exhibited a marked inhibition of reporter translation from the beginning of the CHX treatment (*p* < 0.0001), confirming that no post-translational events occurred to support protein content decay (Figure 5B). Subsequently, ActD treatment of 5′-Ld-SL1-mediated reporter gene expressing cells evidenced the Wuhan-1 Nsp1’s ability to maintain GFP mRNA stability, consistent with its known protective function on viral mRNAs (Figure 5C). Instead, Nsp1 variants carrying the Δ82–85 and Δ82–86 deletions did not show any particular protective activity on the 5′-Ld-SL1-mediated GFP mRNA (Figure 5C). To argue the protective effect of 5′-Ld-SL1-containing mRNA by the Nsp1, immunoblotting was performed on CHX-treated samples. This analysis indicated that the reporter protein translation and turnover remained unaffected over time by CHX treatment of Wuhan-1 Nsp1-expressing cells, as well as those concurrently expressing the Δ82–85 and Δ82–86 Nsp1 variants (Figure 5D). Interestingly, the CHX chase assay revealed a significant destabilizing effect caused by the 82–85/86 deletion on Nsp1 itself. In particular, whereas the Wuhan-1 Nsp1 demonstrated robust stability up to 4 h from CHX treatment, the Δ82–85 and Δ82–86 protein variants exhibited a three-fold reduced stability, within the same timeframe (Figure 5E). Similarly, the deleted Nsp1 proteins exhibited significantly less stability than their corresponding original variants during viral infection and replication in Vero E6 cells. Specifically, CHX treatment of BA.2_Δ82–85- (fold change 0.17 ± 0.04; *p* < 0.0001) and BQ.1.1_Δ82–86- (fold change 0.13 ± 0.01; *p* < 0.0001) infected cells resulted in drastic Nsp1 dropout at 4 h post-treatment (Figure 5F). In contrast, Vero E6 cells infected with the corresponding original lineages showed an increased Nsp1 stability (fold change 0.52 ± 0.12; *p* = 0.01 and 0.57 ± 0.01; *p* = 0.0083, respectively) at 4 h post-CHX treatment (Figure 5F). Therefore, the 82–85/86 Nsp1 N-terminal deletion also affects its stability. Thus, we could speculate that the reduced protein stability of the deleted Nsp1 may affect, at least in part, its diminished protection of viral mRNAs.

### 3.6. The 82–85 Nsp1 Domain Alters Viral mRNAs’ Binding Affinity

**Figure 6:**
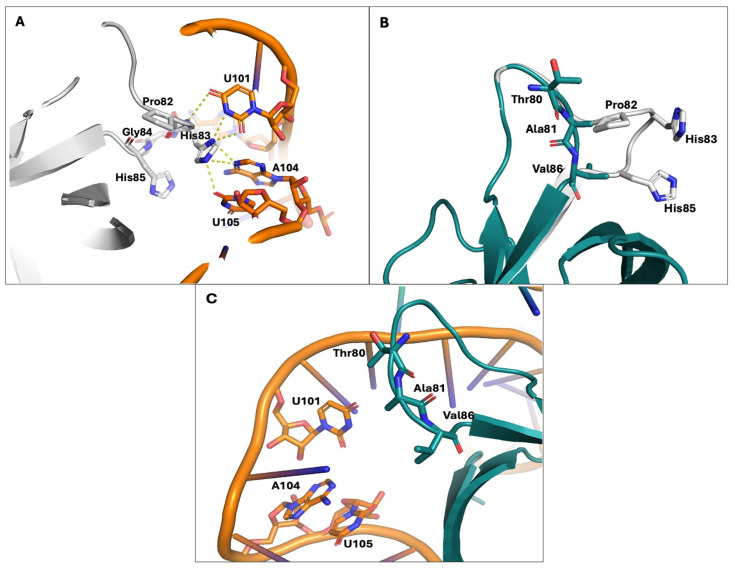
Molecular docking to assess the interaction affinity between deleted Nsp1 and 5′-Ld-SL1 mRNA. (**A**) Graphical representation of the best-scored docked pose of the wt protein (grey, taken from the protein data bank entry 7K7P) and 5′-SL1 mRNA (orange); (**B**) superimposition of the wt (grey) and Δ82–85 variant (deep teal); (**C**) the best-scored docked pose of the Δ82–85 variant (deep teal) and 5′-SL1 mRNA (orange).

Docking calculations were performed to investigate the interactions between Nsp1 and the viral 5′-Ld-SL1 mRNA and to evaluate how deletion of the 82–85 domain could affect mRNA binding. Figure 6A shows that the backbone of Pro82 is involved in a hydrogen bond with U101, while His83 forms a hydrogen bond network with U101, A104, and U105. This interaction pattern confirms the direct role played by sequence 82–85 of Nsp1 in mRNA binding.

Furthermore, superimposing the structures of Wuhan-1 and Δ82–85 proteins reveals differences in spatial and conformational arrangements, suggesting that the shorter loop formed by Thr80 and the hydrophobic amino acids Ala81 and Val86 may engage in different interactions with mRNA (Figure 6B). Although Δ82–85 Nsp1 retains the ability to bind viral mRNA, the deletion of the four amino acids results in the loss of loop interactions with nucleic acid bases (Figure 6C). Consequently, the modified Nsp1 protein exhibits a decreased affinity for mRNA binding compared to Wuhan-1 Nsp1, as supported by the docking scores (Wuhan-1: −299 kcal/mol; Δ82–85: −248 kcal/mol). In summary, molecular docking simulations and superposition of three-dimensional structures of Wuhan-1 and Δ82–85 Nsp1 reveal significantly different abilities to bind viral mRNA, consistent with the reduced capacity of the protein to recognize and translate viral mRNA.

## 4. Discussion

Viruses have developed various mechanisms in order to overcome host defenses and exploit cellular metabolic pathways for their own genome replication and protein synthesis. Among these strategies, virus-induced host protein shut-down is a crucial step for enhancing viral pathogenesis. This phenomenon has been demonstrated for several pathogens, including the Influenza virus and Coronaviruses (CoVs) [40,41,42,43,44]. Simultaneously, viral transcripts must maintain robust expression during host shutoff, which can be achieved through the employment of alternative RNA processing and ribosome recruitment mechanisms [45,46]. In this context, previous studies on SARS-CoV-1 indicated that the Nsp1 viral protein is involved in both viral replication and the innate immune system hindrance. Numerous studies have also investigated the translational repression and the degrading functions of SARS-CoV-2 Nsp1 on cellular mRNAs. However, the mechanisms by which the viral Nsp1 protein coordinates both activities against cellular mRNA while sparing viral transcripts remain largely elusive. Virus variants containing various deletions in the Nsp1 N-terminus have been previously described by Lin et al. [42]. The deletion identified in that study (a.a. 79–89) was likely not important for Nsp1–ribosome binding ability but significantly suppressed the type I IFN response and attenuated virus replication [47]. In this study, we analyzed mutational variations in the SARS-CoV-2 Nsp1 protein through the use of naturally occurring Nsp1 partially deleted virus lineages. Virus strains bearing N- (a.a. 82–85/82–86) or C-terminal (a.a. 141–143) Nsp1 deletions were isolated and tested in vitro by using cell-based assays to evaluate their respective fitness. Despite the considerable number of sequences deposited so far, the global prevalence of the Δ82–85 and Δ82–86 Nsp1-deleted strains is 0.82% and 0.78%, respectively, and they are distributed worldwide. Within the indicated domain, the methionine at position 85 was found to be the most frequently mutated or deleted among all SARS-CoV-2 strains, with almost 2.9% prevalence of changes. Conversely, other residues in the selected domain were less frequently targeted by changes (1.5%), suggesting their reduced impact on protein function (Table S1). Unfortunately, experimental investigation of such mutants was not feasible, as clinical specimens carrying these mutations were identified and isolated for further in vitro analysis. Thus, we could only speculate about their implications in virus fitness. In contrast, a higher prevalence (2.02%) was observed for virus lineages carrying the Δ141–143 deletion. Nonetheless, these deletions were equally reported by Outbreak.info [48] as circulating SARS-CoV-2 lineages or variants. This study may significantly contribute to the knowledge of Nsp1 function in terms of virus gene translation, virus replication, and innate immunity modulation. In comparison to previous studies, we identified a more specific amino-acid sequence (82–85) as critical for Nsp1 function during viral infection and replication. We observed that virus lineages with the N-terminal deletion (Δ82–85 and Δ82–86) exhibited a more pronounced attenuation in virus replication compared to their corresponding original lineage counterparts. This was demonstrated in both type I interferon (IFN-β)-deficient (Vero E6 cells) and competent (Calu-3 cells) cell systems, supporting the evidence that the reduced replication proficiency was not influenced by the antiviral activity of secreted IFN-β. Indeed, despite the markedly reduced progeny virus release in Δ82–85 and Δ82–86 Calu-3-infected cells, a sustained release of IFN-β was observed in original and deleted virus strains, regardless of the strain used. However, the augment secretion of IFN-β by both the BA.2 deleted variants infected Calu-3 cells evidenced the Nsp1 negligible activity towards the cytokine control. Thus, factors other than the Nsp1 protein were involved in the induction of IFN-β in a strain-specific manner, as clearly evidenced by previous reports [2,3,4,5,6,27,28]. The replication efficiency and production of intracellular dsRNAs, for instance, play a central role in pattern-recognition receptors’ (PRRs) activation and downstream IFN-β induction [28]. We then investigated the host shutoff activities of selected deleted Nsp1 proteins. N-terminal domain (NTD)-deleted Nsp1 proteins (amino acid residues 82–85) retained the ability to block the translation of proteins under a human promoter (HBB-GFP) without affecting mRNA abundance. This was observed through molecular investigations using RT-qPCR and immunoblotting for the GFP reporter protein. While the Δ82–85 and Δ82–86 Nsp1 proteins showed an evident reduction in the degrading activity of cellular mRNAs, ribosome recruitment and translational machinery hindrance still occurred, leading to the partial suppression of host protein translation (Figure 4). In addition, molecular docking analysis of deleted Nsp1 and 5′-Ld-SL1 viral mRNAs revealed a reduced capability of the Nsp1 variants to recognize and efficiently bind viral mRNAs (Figure 6). However, the cycloheximide (CHX) chase supported the evidence that the decreased viral replication could be partly attributable to decreased protein stability of deleted Nsp1 rather than enhanced RNA degradation (Figure 5). Indeed, the stability of deleted Nsp1 was found to be altered compared to the parental protein in vitro in both transfected and infected cells, potentially affecting protein function in terms of viral mRNA protection and ribosome binding for translation. Although 5′-Ld-SL1 mRNAs were not degraded, they were neither stabilized nor engaged by ribosomes for efficient translation into viral components. This aspect warrants further investigation to understand the impact of mutated viral proteins on virus fitness. These observations align with the findings of other studies. It appears that the Nsp1 NTD promotes RNA decay, and R_99_ and R_124_/K_125_ residues are believed to play a fundamental role in this mechanism, presumably by recruiting or stabilizing host nucleases at the Nsp1–ribosome–mRNA complex site [11,26,43]. However, it is necessary to further investigate the role of Nsp1. Moreover, the aforementioned mutations rescued cellular mRNA content, confirming that Nsp1 domains responsible for mRNA stability and translation are located in distinct parts of the protein. Unlike cellular mRNAs, viral mRNAs are highly translated in the presence of Nsp1 [46]. For Coronaviruses, including SARS-CoV-2, a conserved stem loop (SL1) within the 5′-UnTranslated Region (5′-UTR) present on viral mRNAs is required for Nsp1-mediated viral gene translation [34,45,46]. Several models have been proposed to explain the escape of viral mRNA from degradation. The most reliable one suggests that viral mRNAs containing the SL1 interact with Nsp1 and, in association with cellular factor(s), induce a conformational change in Nsp1 that unplugs its C-terminal domain from the 40S entry channel, thereby allowing mRNA translation [36,47,48,49]. Specific mutations within NTD of Nsp1 (R_99_A and R_124_A/K_125_A) have negative effects on the translation of SARS-CoV-2 leader mRNA, instead. In particular, these residues are crucial for the SL1-triggered conformational changes to Nsp1 or may prevent proper assembly of this multicomplex that enables viral mRNA translation [11]. In this study, our findings highlight that the Δ82–85 Nsp1 variant can diminish the translation of viral mRNA containing SL1 without impacting its decay but significantly affecting its recognition by ribosomes. This discovery holds significant implications to understand the role of Nsp1 in virus pathogenesis. It suggests that specific Nsp1 mutations or deletions, such as the 82–85 domain, may impair viral pathogenesis due to translational suppression of viral transcripts, although the shutoff of host genes still occurs. Notably, among the eleven Nsp1 N-terminal domain (NTD) amino-acid deletions (a.a. 79–89) previously identified as critical for virus fitness by Lin et al., only four (a.a. 82–85) are associated with the lower viral load. This insight has implications for potential antiviral drugs targeting the Nsp1 protein, particularly the 82–85 region. Despite Nsp1 being a validated target for therapeutic action, there have been few studies conducted on Nsp1 for structure-based drug discovery through in silico screening and identification of potential inhibitors. Only a few studies have been conducted, resulting in the identification of potential antiviral molecules. Among them, Montelukast and Mitoxantrone dihydrochloride have been proven to efficiently bind to the C-terminus of SARS-CoV-2 Nsp1 [50,51].

The Nsp1 N-terminal domain can also be a valid target for antiviral drug design. Specifically, since the N-terminal domain of Nsp1 interacts with the stem-loop 1 (SL1) on the 5′-UTR of viral mRNA, targeting the SL1 could prevent its binding to Nsp1 and, consequently, decrease the viral translation of its mRNAs. Several drugs have been shown to bind the SL1 region, including glycyrrhizic acid, lobaric acid, garcinolic acid, and tirilazad [52,53]. Therefore, our results pave the way for an alternative approach to directly target the N-terminal domain of the viral Nsp1 protein rather than the SL1 genomic elements.

Furthermore, the reported data provide valuable information about Nsp1 activity and functional domains which could be examined to reduce the virus virulence and enhance our knowledge of the proteins that could attenuate the virus.

## 5. Conclusions

In this study, our findings highlight that the 82–85 domain within the N-terminus of the SARS-CoV-2 Nsp1 protein is crucial in inhibiting the translation of viral mRNAs containing the 5′-SL1 sequence, without affecting their decay. This evidence provides new insights into virus biology and carries significant implications for understanding the role of Nsp1 in virus pathogenesis. Our data suggest that specific mutations or deletions of the 82–85 domain of Nsp1 may impair viral pathogenesis by suppressing the translation of viral proteins, in agreement with the observed lower viral load in naturally occurring virus lineages having the indicated Nsp1 deletion. In detail, the deletion of this domain negatively impacts on viral mRNAs recognition by the viral protein and its interaction with ribosomes. This insight paves the way for the development of antiviral drugs targeting the Nsp1 protein. Furthermore, the reported data provide valuable information about Nsp1 activity and functional domains that could be explored to mitigate virus virulence and enhance our knowledge of proteins capable of attenuating the virus.

## 6. Limitation of the Study

The analysis of the correlation of virus variants with clinical profiles of the disease was hindered by the lack of clinical data concerning patients infected with the specific SARS-CoV-2 lineages investigated. This study relied on experiments that serve as a proxy for cellular or viral transcripts.

## Data Availability

Data are contained within the article or Supplementary Material. The data presented in this study are available upon request.

## Supplementary Materials

The following supporting information can be downloaded at: https://www.mdpi.com/article/10.3390/v16050689/s1, Figure S1: Evaluation of host cell shutoff in Nsp1 variants expressing cells; Figure S2: Evaluation of Nsp1 variants modulation of viral genes expression; Table S1: Prevalence of mutational changes in the N- and C-terminal domains of SARS-CoV-2 Nsp1 protein.
