# Supplementary material for: Deletion of 82–85 N-Terminal Residues in SARS-CoV-2 Nsp1 Restricts Virus Replication
Source: Viruses. 2024 Apr 26;16(5):689. doi: 10.3390/v16050689 (PMC11125901; doi:10.3390/v16050689)
Supplement: Supplementary file 1 [file viruses-16-00689-s001.zip › viruses-2972619-supplementary.pdf]

viruses

MDPI

Supplementary Materials

Deletion of 82–85 N-Terminal Residues in SARS-CoV-2 Nsp1 Restricts Virus Replication

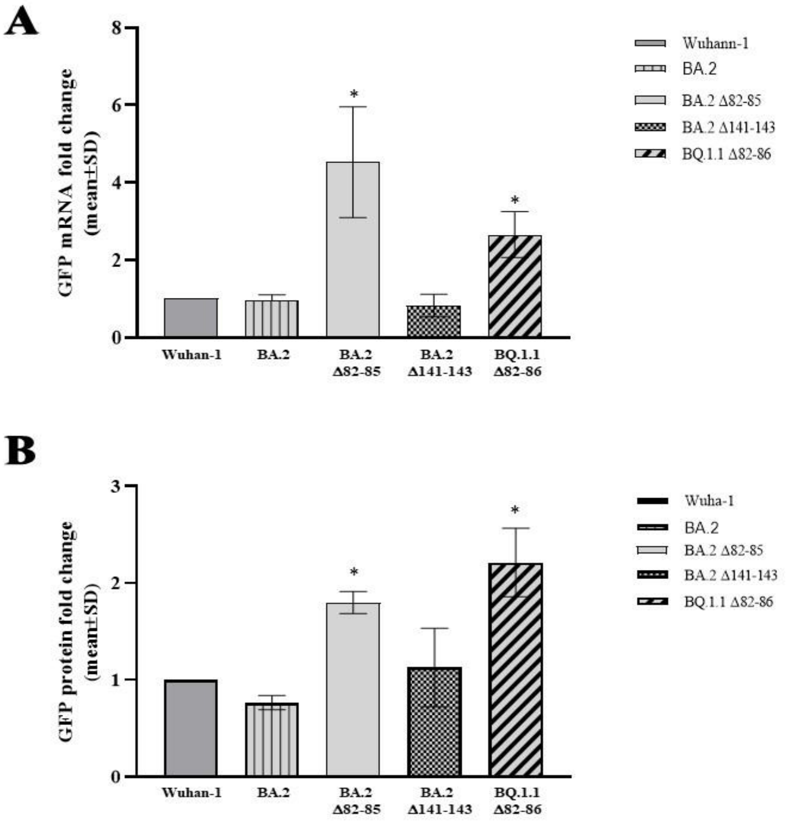

Figure S1. Evaluation of host cell shutoff in Nsp1 variants expressing cells. The impact of Nsp1 deleted mutants on the human beta-globin (HBB)-promoter mediated Green Fluorescent Protein (GFP) was assessed in HEK-293T cells. (A) The transcriptional control of GFP was examined by using total RNA purified from cells transfected with HBB-GFP and Nsp1-expressing plasmids. Cells were collected at 48 hours post-transfection and the specific GFP mRNA content was measured by quantitative reverse-transcription polymerase chain reaction (RT-qPCR). GAPDH gene expression served for relative quantification based on the $2^{-\Delta\Delta Ct}$ method. At least three independent experiments ($n \geq 3$) were conducted, and representative data are presented as mean values ± standard deviations. (B) The modulation of GFP expression in Nsp1 variants expressing HEK-293T cells was also evaluated as protein level through western blotting. Equal amounts of total cell lysates were resolved by SDS-PAGE, and specific antibodies were used for GFP, GAPDH, and Nsp1 proteins, with a representative image provided in the lower panel. Densitometric analysis was conducted using ImageJ software. Graph values are presented as the mean fold change in GFP band intensity ± standard deviations (SD). Significance was as * $p < 0.05$ between each sample and the Wuhan-1 expressing sample.

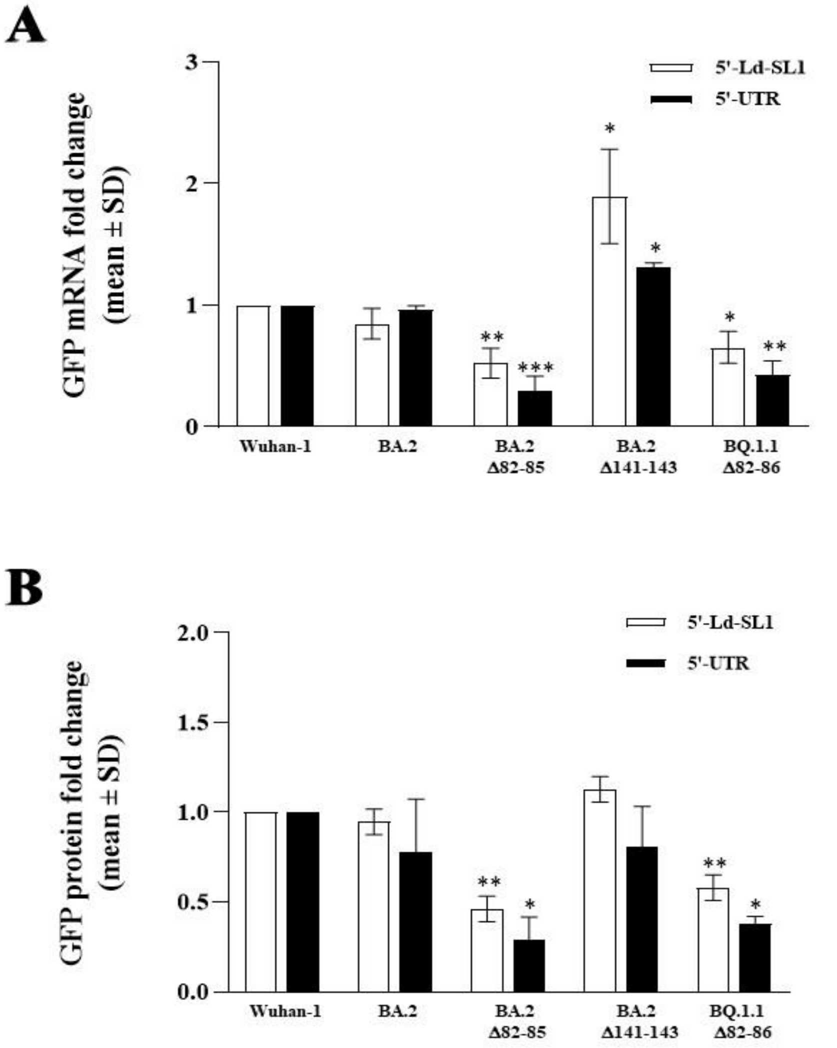

Figure S2. Evaluation of Nsp1 variants modulation of viral genes expression. The impact of Nsp1 deleted mutants on viral 5′-Ld-SL1- or 5′-UTR-mediated GFP expression was evaluated in HEK-293T cells. (**A**) Transcriptional control of GFP was examined in 5′-Ld-SL1- or 5′-UTR-GFP transfected samples in combination with Nsp1-expressing plasmids. Cells were collected at 48 h, and total RNA was purified. GFP and GAPDH mRNAs were quantified by RT-qPCR using the $2^{-\Delta\Delta Ct}$ analysis. Data were presented as mean values ± standard deviations (SD) from different experiments. (**B**) GFP protein content was assessed in Nsp1 variants-expressing HEK-293T cells by Western blotting. Equal amounts of total cell lysates were resolved by SDS-PAGE, and specific antibodies were used to probe for GFP, GAPDH, and Nsp1 proteins. A representative image was provided in the lower panel. Densitometric analysis was performed using ImageJ software. Graph values are presented as the mean fold change in GFP band intensity ± standard deviations (SD). Significance was reported as * $p < 0.05$, ** p <0 .001 and *** $p < 0.0005$ between each sample and the Wuhan-1 expressing sample.

Table S1. Prevalence of mutational changes in the N- and C-terminal domains of SARS-CoV-2 Nsp1 protein.

Change	n° sequences	Total SARS-CoV-2 sequences	prevalence (%)
**G_{82}**	246792	16588391	1.49
**H_{83}**	255533	16588391	1.54
**V_{84}**	273787	16588391	1.65
**M_{85}**	470203	16588391	**2.83**
**V_{86}**	283385	16588391	1.71
**K_{141}**	338522	16588391	**2.04**
**S_{142}**	341032	16588391	**2.06**
**F_{143}**	342487	16588391	**2.06**
**Δ82-83**	233215	16588391	1.41
**Δ82-84**	226133	16588391	1.36
**Δ82-85**	136535	16588391	0.82
**Δ82-86**	130715	16588391	0.79

The amino acid changes within the 82-86 and 141143 domains of the Nsp1 protein were analysed based on the Gisaid deposited sequences. The prevalence was calculated as a percentage of reported cases of single amino acid point mutation or deletion or different combinations of them. In bold, changes with the higher prevalence are indicated, suggesting their minor effects on protein function.
